# New Dental Chair

**Published:** 1877-09

**Authors:** 


### NEW DENTAL CHAIR.

We have had in use for several months S. S. White's New Dental Chair, and find it to answer the requirements much better than any one we have as yet used. It embraces about all the movements requisite for a dental chair, and its adjustments are made with facility and precision.

It is very thoroughly made and its machinery is so simple as not to be easily deranged or broken. A marked degree of perfection and simplicity are combined in the production of this chair.

We regard it as the best chair now in the market. How long it will be before we will have something better, if better can be, time will only reveal.

